# Combining magnetic nanoparticle with biotinylated nanobodies for rapid and sensitive detection of influenza H3N2

**DOI:** 10.1186/1556-276X-9-528

**Published:** 2014-09-26

**Authors:** Min Zhu, Yonghong Hu, Guirong Li, Weijun Ou, Panyong Mao, Shaojie Xin, Yakun Wan

**Affiliations:** 1 The Key Laboratory of Developmental Genes and Human Disease, Ministry of Education, Institute of Life Sciences, Southeast University, Nanjing 210096, People’s Republic of China; 2 Jiangsu Nanobody Engineering and Research Center, Nantong 226010, People’s Republic of China; 3 State Key Laboratory of Materials-Oriented Chemical Engineering, College of Biotechnology and Pharmaceutical Engineering, Nanjing Tech University, Nanjing 210009, People’s Republic of China; 4 302 Military Hospital of China, Beijing 100039, People’s Republic of China; 5 Sipailou No. 2, Southeast University, Nanjing 210096, People’s Republic of China

**Keywords:** Influenza A grade 2 (H3N2), Nanobody, Magnetic nanoparticles, Biotinylation

## Abstract

Our objective is to develop a rapid and sensitive assay based on magnetic beads to detect the concentration of influenza H3N2. The possibility of using variable domain heavy-chain antibodies (nanobody) as diagnostic tools for influenza H3N2 was investigated. A healthy camel was immunized with inactivated influenza H3N2. A nanobody library of 8 × 10^8^ clones was constructed and phage displayed. After three successive biopanning steps, H3N2-specific nanobodies were successfully isolated, expressed in *Escherichia coli*, and purified. Sequence analysis of the nanobodies revealed that we possessed four classes of nanobodies against H3N2. Two nanobodies were further used to prepare our rapid diagnostic kit. Biotinylated nanobody was effectively immobilized onto the surface of streptavidin magnetic beads. The modified magnetic beads with nanobody capture specifically influenza H3N2 and can still be recognized by nanobodies conjugated to horseradish peroxidase (HRP) conjugates. Under optimized conditions, the present immunoassay exhibited a relatively high sensitive detection with a limit of 50 ng/mL. In conclusion, by combining magnetic beads with specific nanobodies, this assay provides a promising influenza detection assay to develop a potential rapid, sensitive, and low-cost diagnostic tool to screen for influenza infections.

## Background

Influenza A and B viruses cause a pandemic threat to human health throughout the world [1]. Sporadic transmission of influenza viruses from birds to humans could lead to unpredictable pandemic outbreaks. Influenza is an infectious disease of the respiratory tract that can infect millions of people and kills hundreds of thousands of them [2]. Humans, infected with influenza A, manifest typically an acute upper respiratory tract illness characterized by fever, cough, and sore throat. Disease severity depends mainly on the virulence of the influenza virus strain and immune competence of the patients [3]. Influenza viruses are members of the Orthomyxoviridae family, and they are further classed as A, B, and C viruses [4]. Until now, 17 influenza A hemagglutinin (HA) subtypes have been described. However, only a limited number of influenza A viruses (IAV), such as H1, H2, H3, H5, H6, H7, and H9, have been implicated with human infection [5]. The high mutational rate of the virus and frequency of interspecies transmission leading to novel virus subtypes will reduce the current vaccine efficacy and make influenza infection highly unpredictable [6,7].

Due to a broad vaccine deficiency, effective, early, and sensitive detection of all subtypes of influenza A viruses is of significant importance to reduce the mortality of influenza infection [8]. Simple equipment for the fast and low-cost detection of influenza viruses can attract great attention, because such a tool could reveal the threat before spreading the disease [9]. Diagnostic methods to detect the influenza virus have been reported in several studies. Isolation and cultivation of viruses in cell culture has been regarded as the golden standard for virus detection [10]. However, this method is laborious and time-consuming, and it will take several days to identify the virus. Besides cultivation assays, enzyme-linked immunosorbent assay (ELISA) using monoclonal antibodies against influenza viral antigens is a good alternative method for the detection of influenza [11]. PCR, another method used for influenza detection, is more specific, more sensitive, and less time-consuming, compared with traditional methods [12]. However, all of the above methods require sophisticated equipment and are not practical in clinical settings. Therefore, the development of a simple, rapid, and sensitive assay to detect influenza remains a challenge.

Antibody-mediated immunoassays are promising tools for the detection of influenza based on their specificity, accuracy, and stability. Camelidae such as camels, llamas, and alpacas have a humoral immune response that has evolved into heavy-chain-only antibodies. Unlike conventional IgGs, the antigen-binding fragment of these heavy-chain antibodies consists of one single domain referred to as VHH or nanobody, with a molecular weight of approximately 15 kD. A nanobody is one of the smallest known antigen-binding antibody fragment. The CDR3 (the third antigen-binding loop) of nanobodies plays a key role in recognizing complicated structures such as pockets and clefts that are usually inaccessible for conventional antibodies [13]. The reduced size, improved solubility, and good stability of the camelid heavy-chain fragments form the basis of a new generation of antibodies for diagnostic applications [14,15]. In our previous study, we have isolated nanobodies against human prealbumin (PA) and successfully applied them into a sensitive flow injection chemiluminescence immunoassay with a detection limit of 0.01 μg/L [16]. In addition, a nanobody with specificity to small caffeine molecules has been used in an ELISA to measure caffeine concentration in beverages [17]. Thus, nanobodies provide great alternatives to conventional antibodies for diagnosis of diseases.

**Figure 1:**
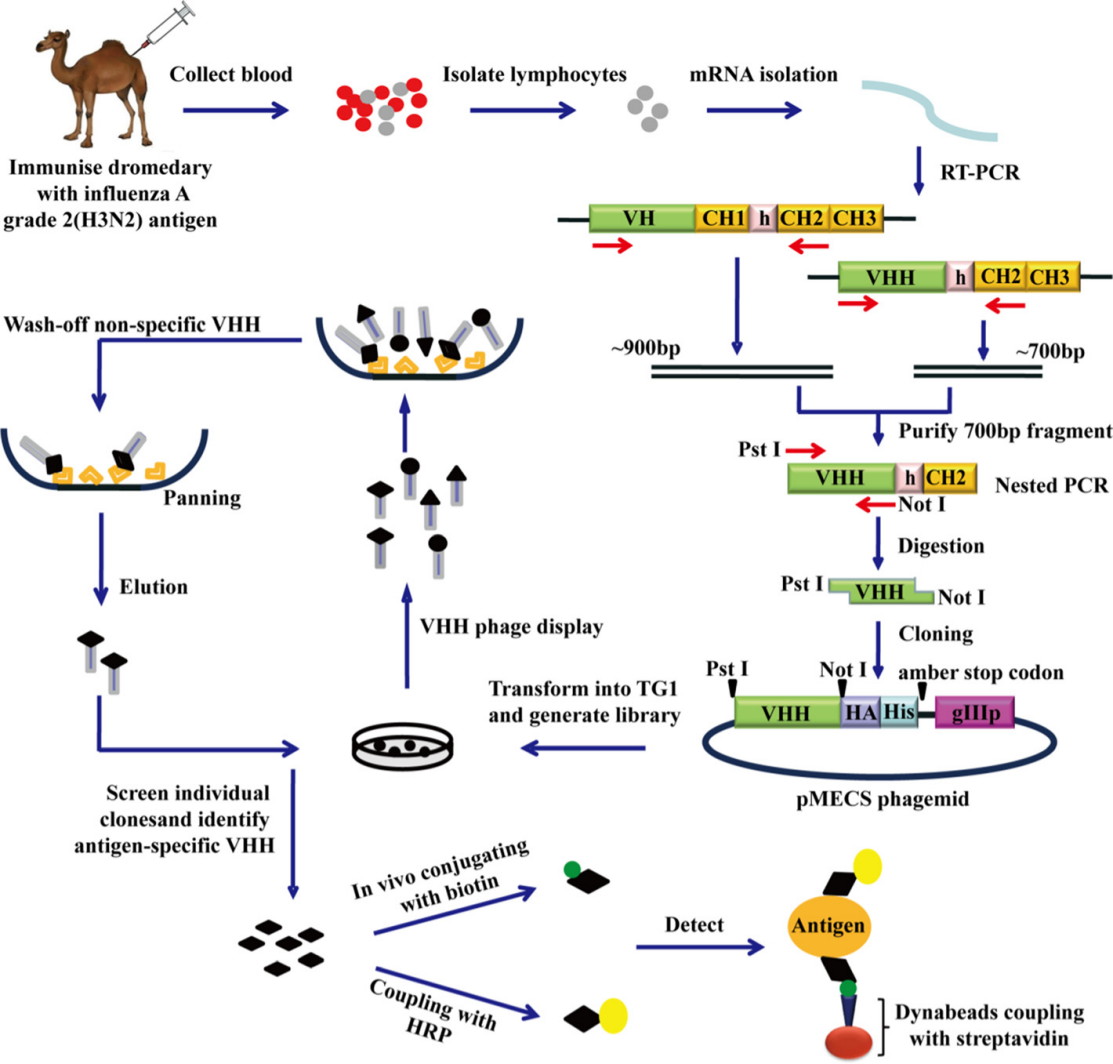
**The whole procedure.** Schematic representation of the strategy to select the influenza A grade 2 (H3N2)-specific single-domain antibodies and to apply the detection approach. The H3N2-specific VHH genes were cloned and selected from an immunized dromedary. Nanobodies identified to recognize different epitopes were used to couple with biotin or HRP to detect antigen.

In this study, we have successfully constructed an immune phage display nanobody library against influenza H3N2. The size of the nanobody library is 8 × 10^8^ transformants with great diversity. After several rounds of biopanning steps, nanobodies specific to H3N2 have been isolated and expressed efficiently in bacterial hosts. Moreover, a novel immunoassay has been developed for the rapid and sensitive detection of influenza H3N2, combining anti-H3N2 Nb3 modified magnetic beads and horseradish peroxidase (HRP)-conjugated Nb1. A sandwich immunoassay was used for capturing and enriching trace amounts of H3N2 and detecting the concentration of H3N2 with relatively high sensitivity and rapidly. The whole procedure is schematically reviewed in Figure 1.

## Methods

### Immunization

A healthy young camel was immunized primarily with pure inactivated influenza A grade 2 (H3N2) virus (1 mL, 100 μg) mixed with an equal volume of Freund’s incomplete adjuvant. This virus was purchased from Microbix Biosystems Inc. (Ontario, Canada) and was inactivated by gamma radiation. To stimulate antigen-specific B cells to raise heavy-chain antibodies, antigen was injected once a week. After seven injections, peripheral blood lymphocytes (PBLs) from 100 mL of blood of the immunized dromedary were isolated by density gradient using Ficoll-Paque™ PLUS (GE Healthcare, Beijing, China) and used to construct the library. All camel experiments were performed according to guidelines approved by Southeast University.

### Construction of the immune single-domain antibody library

Total RNA was isolated from the purified PBLs using the Fast Track 2.0 Kit (Invitrogen, Carsbad, CA, USA) and to calculate the concentration. In order to avoid contamination with VH genes, the variable regions of heavy-chain immunoglobulins (VHH) were amplified by nested PCR. One hundred nanograms of first-strand cDNA was used in each PCR reaction with primers CALL001 and CALL002 (Table 1). This protocol of the first PCR consisted of an initial denaturation step at 94°C for 7 min, followed by 30 cycles of 94°C for 1 min, 55°C for 1 min, and 72°C for 1 min, and a final extension step at 72°C for 10 min. The VHH genes of the first PCR products were analyzed by agarose gel electrophoresis. Gel plugs from the bands near 700 base pairs (bp) were used to extract DNA, used as template in the secondary PCR. This was performed with the primers BACK-1, BACK-2, and PMCF (Table 1) containing restriction enzyme sites *Pst*I and *Not*I, using the same protocol as the first PCR but reduced to 17 cycles. The PCR products were run on 1.5% agarose gel, and the band at approximately 400 bp was extracted from the gel.

**Table 1:** Primers used in this study

**Primer**	**Sequence**
CALL001	5′-GTCCTGGCTGCTCTTCTACAAGG-3′
CALL002	5′-GGTACGTGCTGTTGAACTGTTCC-3′
PMCF	5′-CTAGTGCGGCCGCTGAGGAGACGGTGACCTGGGT-3′
BACK-1	5′-GATGTGCAGCTGCAGGAGTCTGGAGGAGG-3′
BACK-2	5′-GATGTGCAGCTGCAGGAGTCTGGGGGAGG-3′

The amplified second PCR products were digested with *Pst*I and *Not*I restriction enzymes (NEB, Ipswich, MA, USA), then inserted into the phagemid pMECS [18]. Ligation products were transformed into *Escherichia coli* TG1 cells by electroporation. The transformants were plated onto 2 × YT containing 1% glucose and 100 μg/mL ampicillin and cultured at 37°C for 16 h. Plating an aliquot of the library and counting the colony number determined the library size. Many clones were selected randomly and used in a colony PCR to estimate the percentage of clones with a proper insert size within our library.

### Selection of H3N2-specific nanobodies

A representative fraction of the VHH library was cultured and infected with VCSM13 helper phages to express the VHH at the tip of phage particles [18]. Pure inactivated influenza A grade 2 (H3N2) (20 μg) in coating buffer (0.1 M NaHCO_3_, pH 8.2) was used as antigen to coat onto microtiter plates (Nunc Immuno Maxsorp, Roskilde, Denmark) at 4°C overnight. The control was 0.1 M NaHCO_3_ (pH 8.2). After blocking with 0.1% casein in phosphate-buffered saline (PBS) for 2 h and incubation with phage-displayed sdAbs in PBS for 1 h at room temperature, the specific phages were eluted with 100 mM triethylamine, transferred to a fresh tube, and immediately neutralized with 1.0 M Tris-HCl (pH 7.4) and used to infect TG1 cells. This process represented one round of panning. Then, part of the TG1 cells was plated at various dilutions, whereas the remaining of the culture was super-infected with helper phages VCSM13. The generated phage particles were used in the next round of panning. During two to four rounds of panning, the H3N2-specific phages were enriched gradually.

### Periplasmic extract ELISA

To detect the H3N2-specific clones, 95 clones were selected randomly for periplasmic extract ELISA (PE-ELISA). After disrupting the cells by osmotic shock and a centrifugation step (i.e., the periplasmic extract), the nanobodies resided in the supernatant, which was incubated with antigen coated in microtiter plates. This technique is referred to as periplasmic extract ELISA or PE-ELISA. Each clone was cultured in 1 mL Terrific Broth (1 L TB: 12 g peptone, 24 g yeast extract, 4 mL glycerol, 170 mM KH_2_PO_4_, and 0.72 M K_2_HPO_4_) with 100 μg/mL ampicillin, then the expression of VHH by 1 mM isopropyl β-d-1-thiogalactopyranoside (IPTG) was induced. Cells were collected and resuspended into 200 μL TES (0.5 M sucrose, 0.2 M Tris-HCl pH 8.0, 0.5 mM EDTA) for 2 h at 4°C, and 300 μL cold TES/4 was added for 2 h. The supernatant was transferred into the wells of the microtiter plates, in which we have coated inactivated influenza A grade 2 (H3N2) (2 μg/mL). After 1 h, we added mouse anti-HA tag antibody (Santa Cruz Biotechnology, Inc., Santa Cruz, CA, USA) for 1 h and then an anti-mouse IgG-alkaline phosphatase (Sigma-Aldrich, Saint Louis, MO, USA) for 1 h. After washing with PBST (PBS with 0.05% Tween 20) and addition of the chromogenic solution containing bisphosphate (pNPP) (Sigma-Aldrich, Saint Louis, MO, USA), we read the absorbance at 405 nm with an ELISA reader (Bio-Rad iMark™, Bio-Rad Laboratories, Inc., Hercules, CA, USA).

### Expression and purification of selected nanobodies

The selected VHH genes in pMECS were transformed into *E. coli* WK6 electrocompetent cells to express the nanobodies. The cells were grown in TB supplemented with 0.1% glucose, ampicillin (100 μg/mL), and 2 mM MgCl_2_, until absorbance at 600 nm reached between 0.6 and 0.9. The expression of nanobodies was subsequently induced with 1 mM IPTG for 16 h at 28°C. After pelleting the cells, we extracted the periplasmic proteins by osmotic shock. Soluble sdAbs containing His-tags were purified from the cell lysate by immobilized metal affinity chromatography (IMAC) using a His-Select column (Sigma-Aldrich, Saint Louis, MO, USA). After washing with PBS, we eluted the His-tagged proteins with a gradient of increasing concentration of imidazole (pH 7.0) and subsequent dialysis of the fractions of interest into PBS.

### ELISA for nanobody specificity detection

To detect the specificity of nanobodies which we have purified, these nanobodies were tested to combine with several kinds of avian influenza virus by ELISA. Each inactivated influenza virus (5 μg/mL) was coated onto microtiter plates overnight at 4°C in coating buffer. After blocking with 1% bovine serum albumin (BSA) at room temperature for 2 h, 10 μg/mL of nanobodies was added and the plates were incubated at room temperature for 1 h. The following steps were the same as described above for the periplasmic extract ELISA.

We then decided to detect the specificity of these two nanobodies against the H3N2 virus. Hence, 5 μg/mL of recombinant hemagglutinin (HA) protein (Sino Biological Inc., Beijing, China), H3N2 virus, and BSA (control) were coated independently in 96-well microtiter plates, and residual protein binding sites were then blocked with 1% skimmed milk in PBS. After incubation with nanobodies at 10 μg/mL at room temperature for 1 h, anti-His tag mouse monoclonal antibody (Abbkine, Inc., Redlands, CA, USA) was added for 1 h and then anti-mouse IgG-alkaline phosphatase for 1 h. Finally, after adding the substrate, absorbance at 405 nm was read.

### Conjugation with biotin *in vivo*

We subcloned the VHH gene fragment to a pBAD vector after digestion by *Nco*I and *BstE*II restriction endonucleases and co-transformed the ligated material into WK6 cells with pBirA plasmid [19]. The expression of VHH-BAD fusion protein was induced with 1 mM IPTG followed by the addition of 50 μM d-biotin (Bio Basic Inc., Beijing, China) and incubated for 30 min. Cells were collected and the periplasmic proteins extracted by osmotic shock. Soluble nanobodies, which were coupled to biotin, were purified by Streptavidin Mutein Matrix (Roche, Basel, Germany). After washing with PBS, the nanobodies conjugated with biotin were eluted by 6 mM d-biotin and dialyzed into PBS.

### Coupling with HRP

To provide a detection function to the nanobodies, we coupled HRP (Sigma-Aldrich, Saint Louis, MO, USA) to our nanobodies. We added 100 μL fresh NaIO_4_ (0.1 M) into 200 μL HRP (5 mg/mL) for 30 min at 4°C, and 200 μL ethylene glycol (2.5%) was added for 30 min at room temperature. One milliliter of H3N2-specific nanobodies (1 mg/L) was added to incubate overnight at 4°C. Twenty microliters of sodium borohydride (5 mg/mL) was mixed into it for 3 h at 4°C the next day. HRP-conjugated nanobodies were precipitated by saturated ammonium sulfate (SAS). The same volume of SAS was added for 1 h at 4°C and then centrifuged at 10,000*g* for 30 min. Sediments were resuspended in PBS, and the free nanobodies (i.e., non-conjugated to HRP) were removed by ultrafiltration. Then, HRP-labeled nanobodies (Nbs) were dialyzed into PBS. In order to check the efficiency of the enzyme-labeled nanobody, we coated the H3N2 antigen (5 μg/mL, 100 μL) and used the nanobodies coupling with HRP (10 μg/mL, 100 mL) as detector in our ELISA experiment. After adding 3,3′,5,5′-tetramethylbenzidine (Sigma-Aldrich, Saint Louis, MO, USA) to react for 10 min and stopping the reaction with 2 M H_2_SO_4_, the absorbance was measured at 450 nm.

### Double nanobody sandwich method for antigen detection

We used 50 μL Dynabeads M-280 streptavidin (Invitrogen, Carlsbad, CA, USA) for each reaction to capture the Nb3 conjugated with biotin (2 μg/mL) for 30 min. It was washed with PBST for 10 times and 0.1% BSA for 2 times. After blocking with 0.1% BSA for 2 h, serial dilutions of influenza A grade 2 antigen (500 μL) were added for 1 h. The Nb1 coupled with HRP (1 μg/mL) used as a detector was incubated for 45 min followed by washing for 15 times. All of these procedures were performed at room temperature. Finally, we removed the free Nb1-HRP by washing for 25 times using PBST. Then, the visualization was carried out by adding 3,3′,5,5′-tetramethylbenzidine as substrate. After stopping the reaction using 2 M H_2_SO_4_, the readings were measured at 450 nm.

### Temperature sensitivity test

We detected the residual binding capacity of the nanobodies after being incubated at 37°C for different amounts of time. Antigen (10 μg/mL) was coated at 4°C overnight. Each sample has its own blank control, which was coated with NaHCO_3_ (0.1 M, pH 8.2). After blocking with 1% skimmed milk for 2 h, 20 μg/mL Nbs were added to incubate for 1 h. The following steps were the same as described for the periplasmic extract ELISA and ELISA for evaluating the nanobody specificity. The signal with nanobodies that were not incubated at 37°C was considered as 100%.

## Results and discussion

### Construction of immunized phage display nanobody library

**Figure 2:**
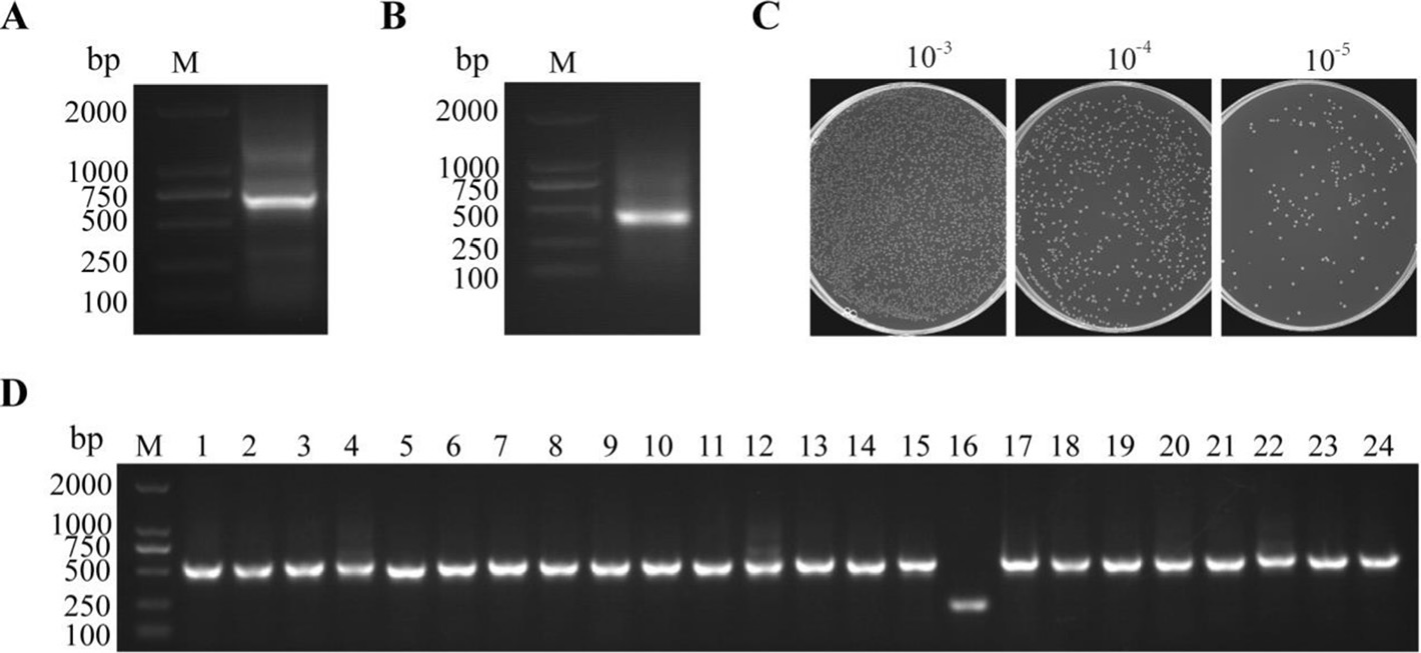
**Library construction. (A)** The segments containing VHH gene fragments were amplified by a first PCR. **(B)** The fragments were amplified by a second, nested PCR. **(C)** Size of the library was determined by counting the number of clones after serial dilutions and plating on plates containing selective antibiotics. **(D)** Clones were randomly selected to detect the percentage of clones with a phagemid containing an insert of a proper size for a VHH.

In order to isolate nanobodies against H3N2 with high affinity and specificity, a 2-year-old healthy Bactrian camel was immunized with the inactivated H3N2 virus over a period of 7 weeks. After the final immunization, we tested the antigen-specific total IgG titer within the serum from the immunized animal and showed that 1:1,000 serum dilution still gave a good signal. This indicated that immunization with inactivated H3N2 has raised a good immune response. According to the procedure in Figure 1, the heavy-chain antibody variable region (also known as VHH) sequences were amplified from lymphocyte cDNA of the camel. Firstly, two PCR products including a 900 bp fragment for VH-CH1-CH2 exons and 600 bp for VHH-CH2 exons (Figure 2A) were amplified with primers CALL001 and CALL002 [20]. The gene for the VHH domain of about 400 bp (Figure 2B) was amplified with nested primers PMCF, BACK-1, and BACK-2 using as template the 600 bp PCR fragment.

For library construction, two restriction enzymes, *Pst*I and *Not*I, were introduced into the 5′ and 3′ ends of the final VHH PCR fragments, respectively. In total, 4.8 μg of purified VHH PCR product and 16 μg of linearized pMECS vector were used for the following ligation. A total of 30 electroporations were performed to transform the ligation mixture into bacterial cells and to obtain a high-quality library with great diversity. The size of the constructed library was calculated from the number of independent colonies on plates and shown to reach 8 × 10^8^ colonies (Figure 2C). This size of the library is about 400 times larger than the one reported previously [21]. It should be a good starting point to retrieve antigen-specific nanobodies. Colony PCR analysis on 24 randomly picked colonies revealed that the percentage of colonies with plasmids having an insert of a proper size for a VHH reached 95% (Figure 2D). These clones have unique sequences after sequencing their VHH fragment. All together, these results demonstrated that we have successfully constructed a phage display nanobody library against H3N2.

### Bio-panning of phage display library against H3N2

**Figure 3:**
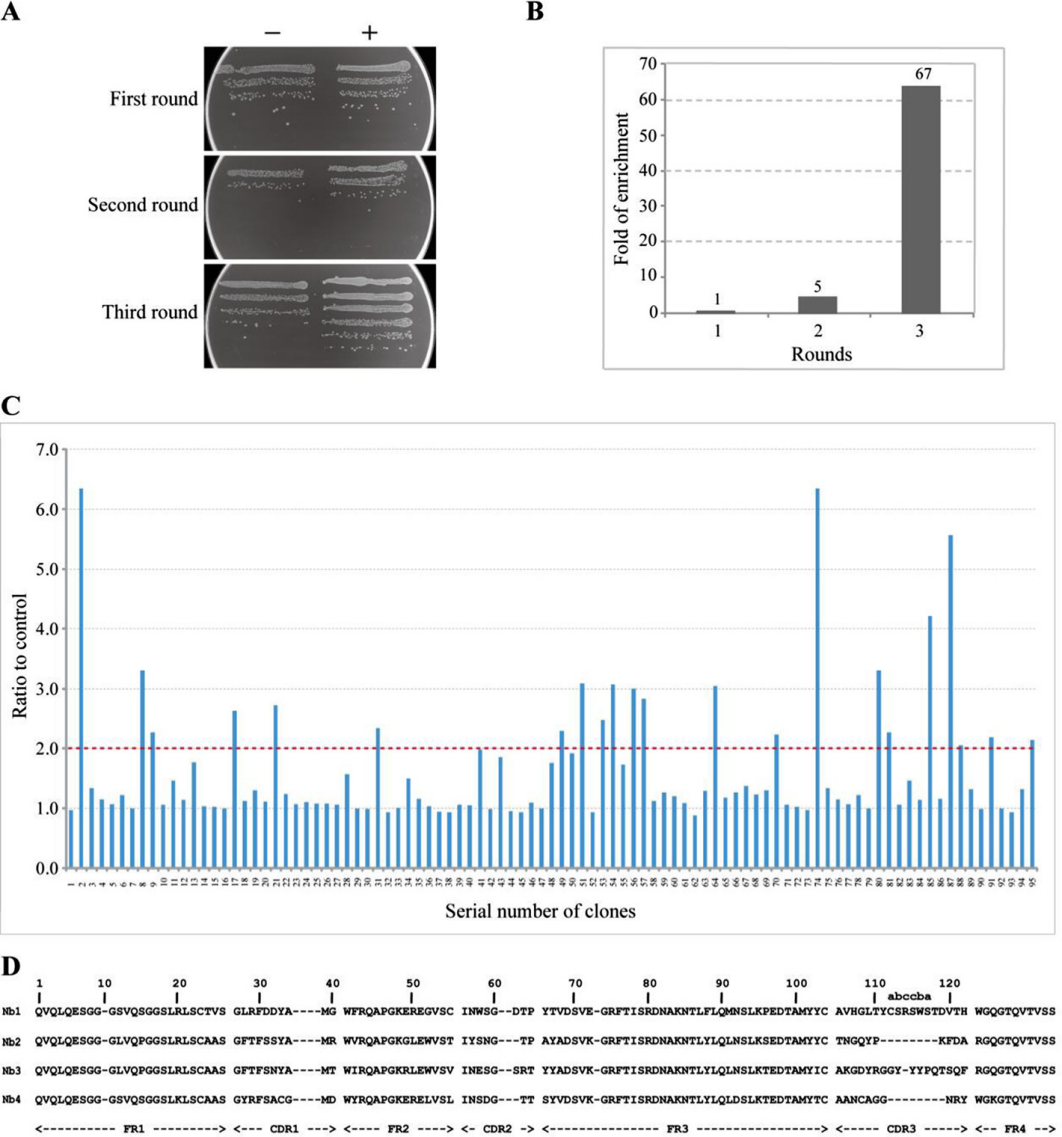
**H3N2-specific VHH genes were selected from the sdAb library. (A)** The enrichment for phage particles in wells coated with antigen versus wells without antigen was detected after each round of panning. Phages collected from each round were incubated with H3N2 virus and NaHCO_3_ (100 mM, pH 8.0), respectively. Then, the eluted phages were transformed into TG1 cells. +: Phages transformed into cells after panning with H3N2 virus. −: Phages panning with NaHCO_3_ were used as control. **(B)** After three rounds of panning, H3N2-specific VHHs were enriched 67-fold compared with control (+/−). **(C)** Periplasmic extract ELISA for 95 colonies. The colonies, whose absorbance signal (nanobodies incubated with H3N2) is more than twofold higher than that for the negative control (nanobodies incubated with NaHCO_3_), were considered as positive. **(D)** Four kinds of different amino acid sequences of anti-H3N2 VHHs were identified.

After getting a good quality of nanobody library for phage display, we performed bio-panning to isolate nanobodies against inactivated H3N2. A total of around 2 × 10^11^ phage particles from the library were used in pannings. In order to evaluate the enrichment during the process of panning, we compared the ratio of colony numbers between pannings on H3N2 influenza viruses and PBS as negative control. As demonstrated in Figure 3A,B, after two and three rounds of biopanning, the ratios have been gradually increased from 5-fold to 67-fold. Based on our experience, high enrichment in only three rounds of biopanning from a large and diverse phage library is important for a successful retrieval of antigen-specific clones [16,22].Next, we have randomly picked 95 colonies after panning and tested whether the PE-ELISA would reveal the presence of nanobodies binding to inactivated H3N2. Of the 95 colonies, 19 showed a good target recognition with binding ratios relative to a non-coated well of more than 2 (Figure 3C). VHH fragments from these 19 colonies were further amplified by PCR and sequenced to confirm the presence of nanobodies. Based on sequencing data, all 19 colonies contained the correct (in frame) VHH fragments. Sequence analysis further classified these 19 nanobodies into 4 classes, which are shown by diversified complementarity determining region 3 (CDR3) sequences (Figure 3D). We define these four classes of nanobodies as Nb1, Nb2, Nb3, and Nb4, respectively.

### Expression of soluble nanobodies and specificity test

**Figure 4:**
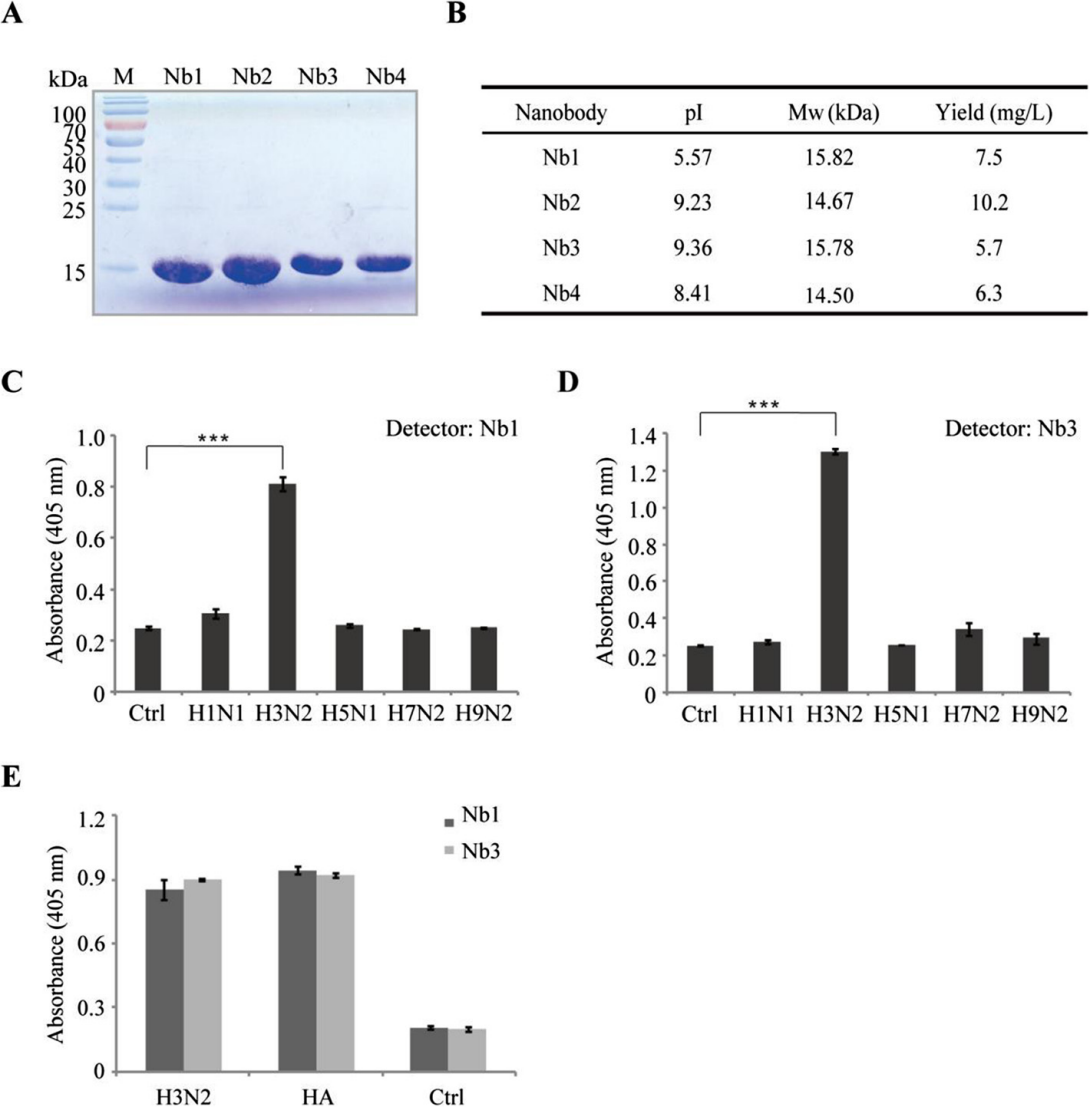
**Purification of H3N2-specific nanobodies and specificity of two paired nanobodies. (A)** Four nanobodies having different sequences were purified by immobilized metal affinity chromatography (IMAC) using a His-Select column. **(B)** Isoelectric point, molecular weight, and yield of four nanobodies. **(C, D)** The Nb1 and Nb3, recognizing two different epitopes as shown via a pairing experiment, were chosen to detect the specificity to five types of different viral proteins by ELISA. One hundred microliters of each inactivated influenza virus (5 μg/mL) was coated onto microtiter plates, and 100 μL nanobodies (10 μg/mL) were added. After reaction with mouse anti-HA tag antibody and then anti-mouse IgG-alkaline phosphatase, the chromogenic solution containing bisphosphate was added, and the absorbance at 405 nm was measured by an ELISA reader. BSA (5 μg/mL) was used as control. Values were the means of three replicates. The values of Nb1 and Nb3 were significantly different from the control (***P < 0.001). **(E)** Specificity of the nanobodies to the proteins on the surface of the H3N2 virus by ELISA. The H3N2 virus and HA protein were coated, and BSA was used as control. After incubation with Nb1 and Nb3, respectively, anti-His tag mouse monoclonal antibody was added, and then anti-mouse IgG-alkaline phosphatase was added for detection. Finally, absorbance at 405 nm was read.

In order to express nanobodies in *E. coli*, the common methods consist in subcloning VHH fragments from the phage display phagemid vector into an expression plasmid. However, in our study, we will use *E. coli* WK6 strains to express the nanobodies. These cells have unique properties which cannot suppress the amber stop codon between gene III and VHH from plasmid pMECS. In this case, we can directly transform the VHH cloned in phage display phagemid pMECS vector from TG1 to WK6 cells to express nanobodies without the need for a subcloning step. Upon IPTG induction, soluble nanobodies are expressed in the periplasmic region of WK6 cells. The induced nanobodies were further purified by Ni^2+^-IDA column. Sodium dodecyl sulfate polyacrylamide gel electrophoresis (SDS-PAGE) analysis of four purified nanobodies showed a good quality with more than 90% purity after one-step purification (Figure 4A). Yields of our four purified nanobodies ranged from 5 to 10 mg/L TB medium (Figure 4B).These four individual nanobodies showed great diversity based on CDR3 sequences, and they may recognize different epitopes of influenza H3N2. Next, we have performed a nanobody-pairing assay with these four nanobodies. It turned out that Nb1 and Nb3 showed to be a great combination for further diagnostic applications based on sandwich immunoassay. As influenza A viruses have been classified into various subtypes, we want to know whether the isolated Nbs from our library are specific to H3N2. We then coated five different subtypes of influenza A viruses H1N1, H3N2, H5N1, H7N2, and H9N2 onto ELISA plates. Interestingly, Nb1 and Nb3 can only recognize H3N2 (Figure 4C,D). In order to assure which kind of protein on the surface of the H3N2 virus is recognized by these nanobodies, we detected its specificity by ELISA. The result in Figure 4E showed that Nb1 and Nb3 were specific to the hemagglutinin protein of H3N2 virus particles. To our knowledge, this is the first report for the identification of nanobodies against influenza H3N2. These nanobodies might be a promising tool to be used for sensitive detection of influenza H3N2.

### Expression and purification of biotinylated nanobodies

Magnetic particle-based immunoassay is a promising tool for clinical diagnosis [23]. In this method, antibody will be conjugated onto magnetic beads and used as a carrier to capture the analytes from the solution. The magnetic beads are easy to manipulate by using an external magnetic field for only a very short period of time. However, the chemical conjugation of antibodies to magnetic particles is usually not directional. Therefore, the covalent conjugation will lead to a decreased binding efficacy of the antibodies. In order to overcome this problem, we decided to label our nanobodies with biotin based on an *in vivo* labeling. Streptavidin-coated magnetic beads will incubate with biotin-labeled nanobodies to efficiently capture the antigens in a directional orientation. In this case, the nanobody conjugated on magnetic particles will be fully functional.

**Figure 5:**
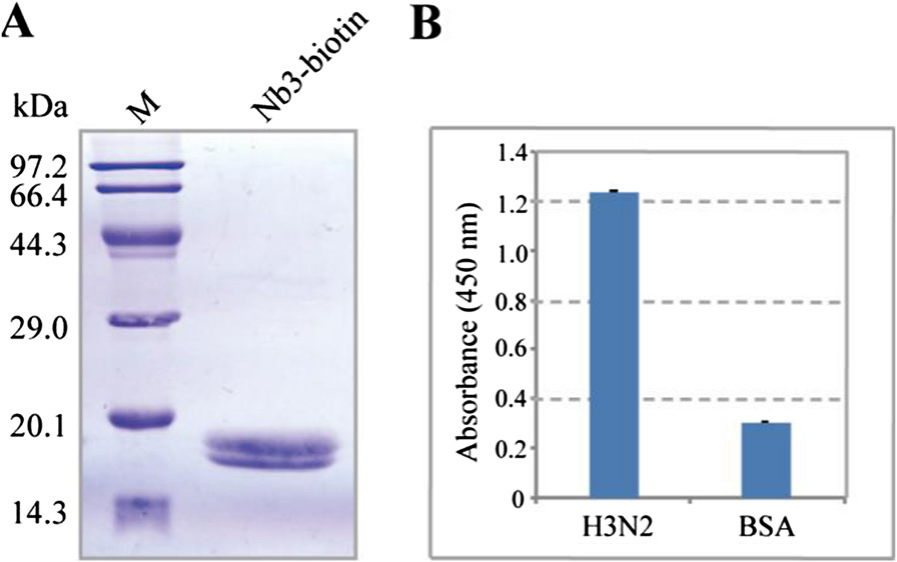
**Purification of Nb3 conjugating with biotin and detection of Nb1 coupling with HRP. (A)** The Nb3 conjugated with biotin was purified on a streptavidin-containing column. **(B)** Analysis of the Nb1 coupled with HRP by ELISA. H3N2 antigen was coated onto the plate directly. Nb1, coupled with HRP, was used as a detector. After stopping the reaction, the readings were measured at 450 nm.

In order to label nanobody with biotin *in vivo* (Additional file 1: Figure S1), we took advantage of the pBAD17 plasmid containing a biotin acceptor domain preceded by an IgA hinge, downstream of the Nb1 and Nb3 sequences. The plasmid was co-transformed with the BirA plasmid (encoding for a biotin protein ligase) into WK6 cells for expression. Nanobodies were extracted from the periplasm of cells by osmotic shock as described previously [20]. Biotinylation nanobodies were further purified on a Streptavidin Mutein Matrix. Finally, biotinylated Nb3 was produced at a final yield of 0.45 mg/L *E. coli* culture (Figure 5A). However, for an unknown reason, we fail to obtain biotinylated Nb1 following the same procedure. As shown in Figure 5B, after coupling with HRP, Nb1 was still able to recognize inactivated influenza H3N2.

### Magnetic nanoparticle-based sandwich immunoassay for rapid detection of influenza H3N2

**Figure 6:**
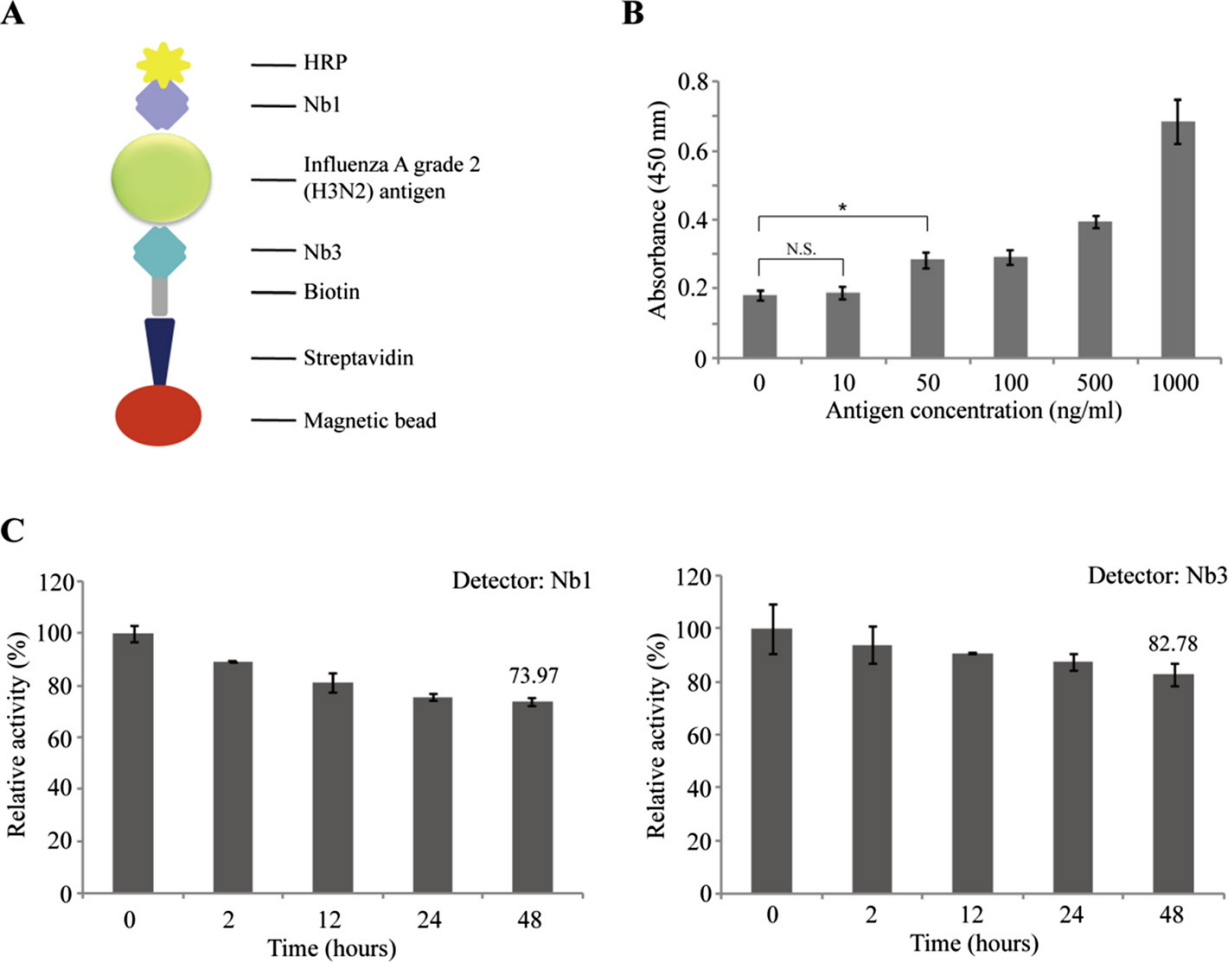
**The use of double nanobody sandwich method for influenza A grade 2 detection. (A)** We used the magnetic beads linked with streptavidin to capture Nb3-biotin and used the Nb1 coupled to HRP as detector for H3N2 antigen detection. **(B)** Serial concentrations of H3N2 antigen were used for their detection. The lowest antigen concentration that could be detected was 50 ng/mL (*P* < 0.05). **(C)** The relative activity of Nb1 and Nb3 which were incubated at 37°C for different amounts of time. The activity of Nb1 and Nb3 reduced 26.03% and 17.22%, respectively, after heat incubation for 48 h.

Biotinylated nanobodies were attached to the magnetic beads via a streptavidin linker. They will be used to capture influenza viral particles. After separation using a magnetic field, HRP-labeled Nb1 was added to detect H3N2. HRP/H_2_O_2_ catalyzed the oxidation of TMB to form a blue-colored complex product changing to yellow after the addition of sulfuric acid to the reaction media [24]. The optical density (OD) value of the product was measured to analyze the concentrations of H3N2 in the reaction. The whole sandwich ELISA immunoassay is summarized in Figure 6A. With the increased concentrations of influenza H3N2, the binding ratio relative to BSA (negative control) also increased. As shown in Figure 6B, the OD value of the sample with 50 ng/mL H3N2 was significantly higher than that for the BSA alone (negative control) (*P* < 0.05). However, 10 ng/mL of H3N2 did not show significant difference from the control (*P* > 0.05). These results demonstrated that the detection limit in our indirect ELISA assay was about 50 ng/mL, which is more sensitive than conventional double-antibody sandwich enzyme-linked immune sorbent assay (DAS-ELISA). Finally, we checked the stability of two H3N2 nanobodies during different incubation times at 37°C. As shown in Figure 6C,D, after 48 h of heat incubation, Nb1 and Nb3 keep 73.49% and 82.78% of their original antigen-binding activity, respectively. We then investigated whether the decreased signal upon incubation at 37°C is due to inactivation of the antigen-binding capacity of the nanobody or due to degradation of the detection tag. To this end, we analyzed the samples incubated for different amounts of time at an elevated temperature in SDS-PAGE and Western blot. The result of the Western blot (Additional file 1: Figure S2) showed that the detection tag was still present on these nanobodies. Thus, we considered that the decreased signal upon incubation at 37°C is due to the decreased antigen-binding activity of nanobodies. Overall, these data demonstrated that Nbs to H3N2 showed good stability and they will provide promising diagnostic materials for clinical application.

## Conclusions

We developed a rapid and sensitive immunoassay for influenza H3N2 detection based on single-domain antigen-binding fragments of camelid-like heavy-chain antibodies. In this study, an immune phage-displayed nanobody library is constructed, from which four classes of nanobodies with good affinity and specificity against H3N2 are successfully isolated and expressed. In addition, Nb3 coupled to magnetic beads and HRP-conjugated Nb1 have been used for rapid and sensitive detection of influenza H3N2. This assay holds a great promise in providing a potentially universal method for detecting all subtypes of influenza viruses.

## Competing interests

The authors declare that they have no competing interests.

## Authors’ contributions

MZ and GL carried out the experiments and participated in the drafting of the manuscript. YH, WO, and PM participated in the detection analysis. SX participated in the design of the study. YW conceived and designed the study and wrote the manuscript. All authors read and approved the final manuscript.

## Supplementary Material

Additional file 1 **Supporting information. Figure S1.** Schematic overview of strategies to conjugate with biotin *in vivo.* H3N2-specific VHH gene was subcloned to the pBAD plasmid, which contained the coding gene of the biotin accepter domain. After being co-transformed into *E. coli* with another plasmid pBirA, the BirA ligase produced by the BirA gene could catalyze free biotin to combine with the VHH-BAD fusion protein. Then, the purified VHH-BAD-biotin complex could be purified. **Figure S2.** Nanobodies were not degraded after heat treatment. The two nanobodies incubated at 37°C after 0, 2, 4, 12, 24, and 48 h were analyzed by SDS-PAGE (A, B) and Western blot (C, D). Click here for file
