# Supplementary material for: Combining magnetic nanoparticle with biotinylated nanobodies for rapid and sensitive detection of influenza H3N2
Source: Nanoscale Res Lett. 2014 Sep 26;9(1):528. doi: 10.1186/1556-276X-9-528 (PMC4199786; doi:10.1186/1556-276X-9-528)

**Additional file 1**

**Figure S1. Schematic overview of strategies to conjugate with biotin *In vivo.*** H3N2-specific VHH gene was subcloned to pBAD plasmid, which contained the coding gene of biotin accepter domain. After co-transformed into *E.coli* with another plasmid pBirA, the BirA ligase produced by BirA gene could catalyze free biotin to combine with VHH-BAD fusion protein. Then the purified VHH-BAD-Biotin complex could be purified.

**Figure S2. Nanobodies were not degraded after heat treatment.** The two nanobodies incubated at 37 ◦C after 0 h, 2 h, 4 h, 12 h, 24 h, 48 h were analyzed by SDS-PAGE **(A, B)** and Western blot **(C, D)**.

**Figure S1**


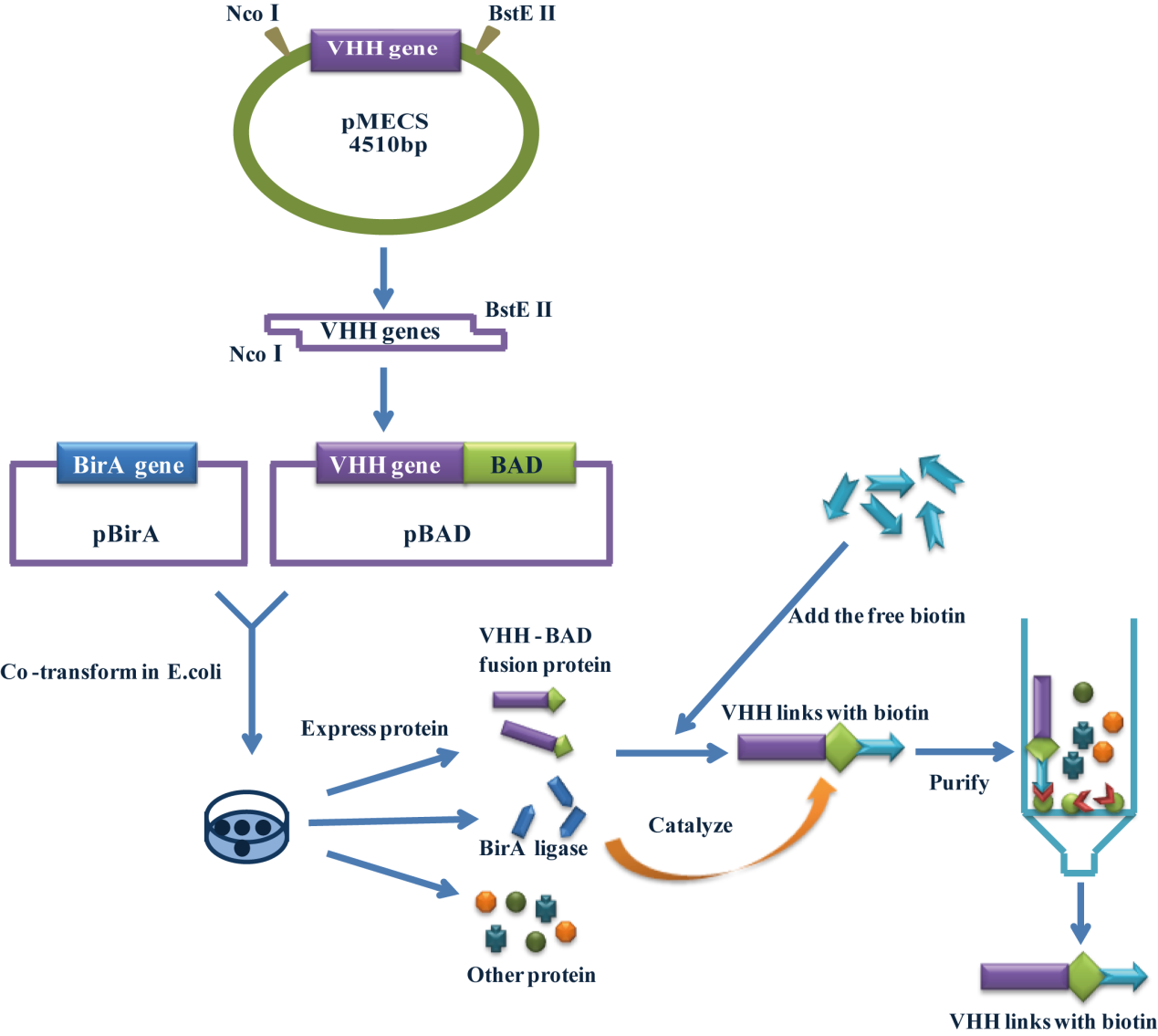


**Figure S2**


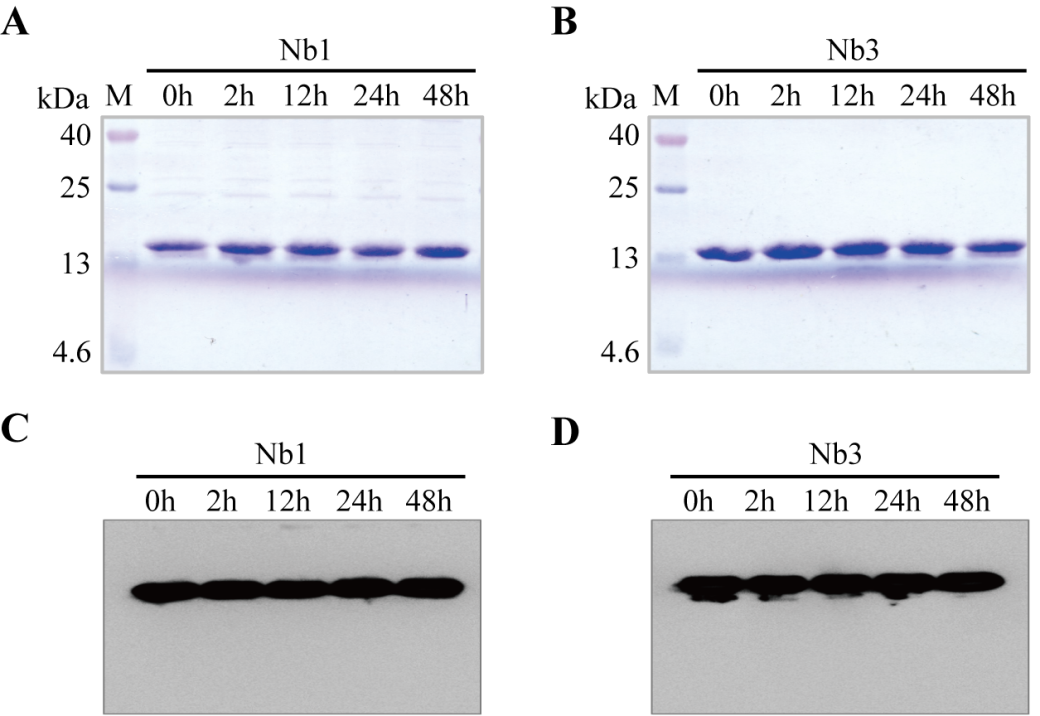

Supplement: Additional file 1 — Supporting information. Figure S1. Schematic overview of strategies to conjugate with biotin in vivo. H3N2-specific VHH gene was subcloned to the pBAD plasmid, which contained the coding gene of the biotin accepter domain. After being co-transformed into E. coli with another plasmid pBirA, the BirA ligase produced by the BirA gene could catalyze free biotin to combine with the VHH-BAD fusion protein. Then, the purified VHH-BAD-biotin complex could be purified. Figure S2. Nanobodies were not degraded after heat treatment. The two nanobodies incubated at 37°C after 0, 2, 4, 12, 24, and 48 h were analyzed by SDS-PAGE (A, B) and Western blot (C, D). [file 1556-276X-9-528-S1.docx]
